# Crystallographic Analysis and Mimicking of Estradiol Binding: Interpretation and Speculation

**DOI:** 10.1289/ehp.1307987

**Published:** 2014-04-01

**Authors:** Thomas G. Osimitz, Michael L. Dourson, A. Wallace Hayes, Sam Kacew

**Affiliations:** 1 Science Strategies, Charlottesville, Virginia, USA; 2 Toxicology Excellence for Risk Assessment (TERA), Cincinnati, Ohio, USA; 3 Harvard School of Public Health, Boston, Massachusetts, USA; 4 McLaughlin Centre for Population Health Risk Assessment, University of Ottawa, Ottawa, Ontario, Canada

In their recent article, Gosavi et al. (2013) presented the results of a crystallographic analysis of the binding of tetrabromobisphenol A (TBBPA) and 3-hydroxy-2,2´,4,4´-tetrabromodiphenyl ether (3-OH-BDE-47) to estrogen sulfotransferase (SULT1E1). The authors demonstrated that the tested molecules fit into the same binding pocket as estradiol. However, although the study’s methodology and interpretation of the crystallographic analysis provide insight into how binding might occur in isolated and *in vitro* systems, they did not provide evidence that the tested molecules would initiate any biological activity with the relevant estrogen receptors (ERs) or proteins in a human body. For example, the ability of TBBPA to interact with the ER and estrogen-related receptors has been evaluated in recombinant yeast strains, mammalian cell–based assays, and tests developed by the Organisation for Economic Co-operation and Development (Lee et al. 2012; Nakagawa et al. 2007; Ogunbayo et al. 2007, 2008; Reistad et al. 2005, 2007; Strack et al. 2007). Those studies found that TBBPA either did not interact with ERs or that it acted as a weak ER agonist/antagonist with a potency orders of magnitude below that of natural ER ligands. In addition, the data presented by Gosavi et al. (2013) did not include the use of controls to validate the methods. The use of both positive controls (such as diethylstilbestrol and ethinyl estradiol) and a negative control (such as testosterone) would provide validation of the analysis and allow for the quantification and comparison of the two test substances in relation to the binding potentials of the controls.

The authors also speculated about the possible additivity of the various brominated flame retardants and their metabolites and suggested that low-dose exposure to multiple low-affinity binding compounds may result in endocrine disruption. However, none of the data presented directly addressed this point.

It is highly complex, not well understood, and speculative to extrapolate data on inhibition of enzymes such as SULT1E1 in *in vitro* assay systems to endocrine-system modulation of selective gene expression, receptor binding, and activation and the production of adverse effects that would characterize endocrine disruption *in vivo* by additivity of different chemicals competing on the same receptors. Only through a more complete understanding of target tissue dosimetry, potency of interaction of the chemical of interest with the macromolecule of interest (e.g., SULT1E1), and subsequent events can one address the likelihood of *in vivo* additivity.

## References

[r1] Gosavi RA Knudsen GA Birnbaum LS Pedersen LC 2013 Mimicking of estradiol binding by flame retardants and their metabolites: a crystallographic analysis. Environ Health Perspect 121 1194 1199; 10.1289/ehp.1306902 23959441 PMC3801471

[r2] Lee HK, Kim TS, Kim CY, Kang IH, Kim MG, Jung KK (2012). Evaluation of *in vitro* screening system for estrogenicity: comparison of stably transfected human estrogen receptor-α transcriptional activation (OECD TG455) assay and estrogen receptor (ER) binding assay.. J Toxicol Sci.

[r3] Nakagawa Y, Suzuki T, Ishii H, Ogata A. (2007). Biotransformation and cytotoxicity of a brominated flame retardant, tetrabromobisphenol A, and its analogues in rat hepatocytes.. Xenobiotica.

[r4] Ogunbayo OA, Jensen KT, Michelangeli F (2007). The interaction of the brominated flame retardant: tetrabromobisphenol A with phospholipid membranes.. Biochim Biophys Acta.

[r5] Ogunbayo OA, Lai PF, Connolly TJ, Michelangeli F (2008). Tetrabromobisphenol A (TBBPA), induces cell death in TM4 Sertoli cells by modulating Ca^2+^ transport proteins and causing dysregulation of Ca^2+^ homeostasis.. Toxicol In Vitro.

[r6] Reistad T, Mariussen E, Fonnum F. (2005). The effect of a brominated flame retardant, tetrabromobisphenol-A, on free radical formation in human neutrophil granulocytes: the involvement of the MAP kinase pathway and protein kinase C.. Toxicol Sci.

[r7] Reistad T, Mariussen E, Ring A, Fonnum F. (2007). In vitro toxicity of tetrabromobisphenol-A on cerebellar granule cells: cell death, free radical formation, calcium influx and extracellular glutamate.. Toxicol Sci.

[r8] Strack S, Detzel T, Wahl M, Kuch B, Krug HF (2007). Cytotoxicity of TBBPA and effects on proliferation, cell cycle and MAPK pathways in mammalian cells.. Chemosphere.

